# Crystal structure of (*E*)-furan-2-carbaldehyde *O*-benzoyloxime

**DOI:** 10.1107/S2056989017011562

**Published:** 2017-08-08

**Authors:** Yousef M. Hijji, Rajeesha Rajan, Said Mansour, Hamdi Ben Yahia

**Affiliations:** a Department of Chemistry and Earth Sciences, Qatar University, PO Box 2713, Doha, Qatar; b Qatar Environment and Energy Research Institute, Hamad Bin Khalifa University, Qatar Foundation, PO Box 34110, Doha, Qatar

**Keywords:** crystal structure, oxime, 2-furan­aldoxime, benzoyloxime ester, hydrogen bonding

## Abstract

In the title compound, C_12_H_9_NO_3_, the benzoate and furan rings are almost coplanar, making a dihedral angle of 11.68 (9)°. The twist angle between the –COO group and the benzene ring is only 2.79 (16)°. In the crystal, mol­ecules are linked by C—H⋯O hydrogen bonds, forming chains along [100]. The mol­ecules stack in a herringbone fashion and inversion-related chains are linked by offset π–π inter­actions [inter­centroid distance = 3.931 (1) Å], forming ribbons propagating along the *a*-axis direction.

## Abstract 2

In the title oxime ester, the benzoate and furan rings are almost coplanar, making a dihedral angle of 11.68 (9)°. In the crystal, mol­ecules are linked by C—H⋯O hydrogen bonds, forming chains along the *a*-axis direction.

## Chemical context   

Oxime esters have shown potencies for inhibiting lipoprotein-associated phospho­lipase A2 (Lp-PLA2) activity. Their derivatives are used for the prevention and treatment of cardiovascular disease (Jeong *et al.*, 2013 ▸, 2006 ▸). These compounds are good anti­oxidants and are used in pharmaceutical compositions for their anti-microbial activity (Liu *et al.*, 2008 ▸; Harini *et al.*, 2012 ▸; Ahluwalia *et al.*, 2017 ▸). In view of this inter­est, we have synthesized the title oxime ester derivative and report herein on its crystal structure.
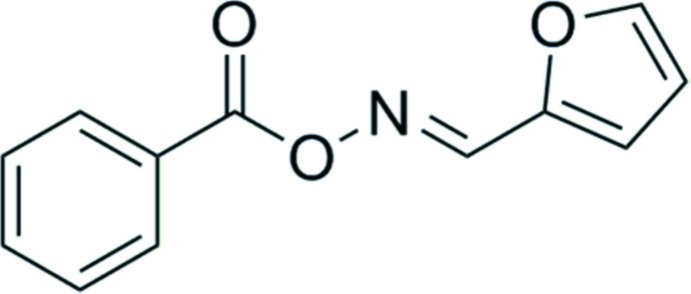


## Structural commentary   

**Figure 1:**
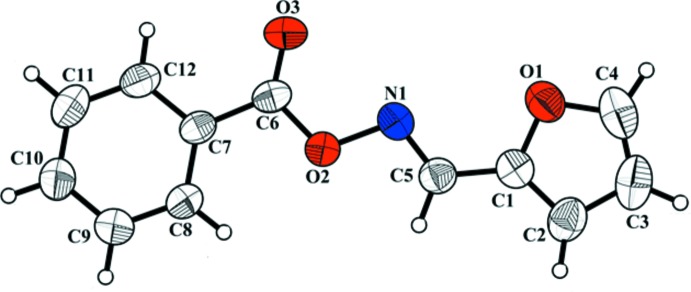
View of the mol­ecular structure of the title compound, with the atom labelling and 50% probability displacement ellipsoids.

The mol­ecular structure of the title compound is shown in Fig. 1 ▸. An intra­molecular short contact (C8—H8⋯O2) is present (Table 1 ▸), which may prevent the –COO group from tilting, since the twist angle between the –C6/O2/O3 unit and the benzene ring (C7–C12) is only 2.79 (16)°. This also might be the reason why the mol­ecule is almost planar. The dihedral angle between the furan (O1/C1–C4) ring and the benzene ring is 11.68 (9)°. The C6—O2 and C6=O3 distances of 1.352 (2) and 1.195 (2) Å, respectively, are typical values for single and double C-O bonds. This overall geometry is very similar to that observed for *E*-benzaldehyde *O*-benzoyl­oxime (Altinbas *et al.*, 2004 ▸). Within the five-membered furan ring, the inter­atomic O1—C1 and O1—C4 distances of 1.369 (2) and 1.367 (2) Å, respectively, are typical values for O—C*sp* ^2^ bonds. The short C4—C3 and C1—C2 bond lengths of 1.324 (4) and 1.347 (3) Å, respectively, and the stretched C2—C3 bond distance of 1.408 (2) Å are typical values observed for double C=C and single C—C bonds, respectively. The –C5/N1/O2 group is twisted by 4.40 (13) ° with respect to the furan ring. The N1—O2 distance of 1.444 (1) Å is only slightly longer than reported in other oxime compounds (Wetherington & Moncrief, 1973 ▸), whereas the C=N—O angle of 106.73 (11)° is slightly smaller.

## Supra­molecular features   

**Figure 2:**
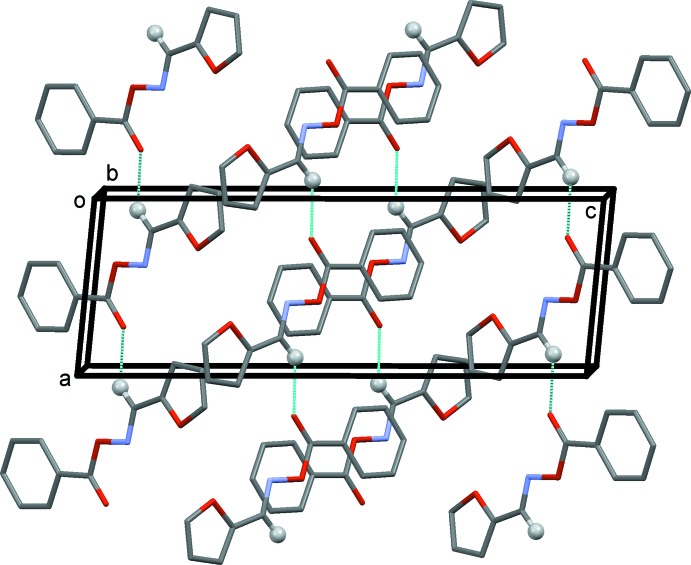
A view along the *b* axis of the crystal packing of the title compound. The C—H⋯O hydrogen bonds, linking mol­ecules to form chains along [100], are shown as dashed lines [see Table 1 ▸; only H atom H5 (grey ball) has been included].

**Figure 3:**
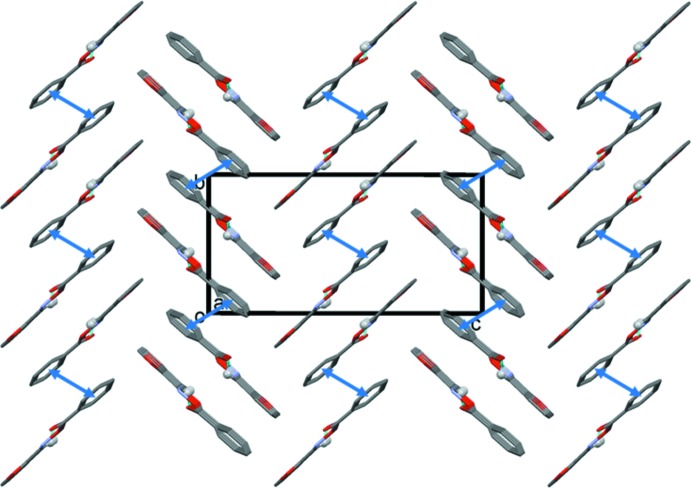
A view along the *a* axis of the crystal packing of the title compound. The offset π–π inter­actions are shown as blue double arrows, and only H atom H5 (grey ball) has been included.

In the crystal, mol­ecules are linked by C—H⋯O hydrogen bonds, forming chains along the *a*-axis direction (Table 1 ▸ and Fig. 2 ▸). The mol­ecules stack in a herringbone fashion and inversion-related chains are linked by offset π–π inter­actions [*Cg*1⋯*Cg*1^i^ = 3.931 (1) Å, inter­planar distance = 3.574 (1) Å, slippage = 1.64 Å, α = 0.03 (7)°, *Cg*1 is the centroid of the benzene ring (C7–C12); symmetry code: (i) −*x* + 1, −*y* + 2, −*z*], forming ribbons propagating along the *a*-axis direction (Fig. 3 ▸).

## Database survey   

A search of the Cambridge Structural Database (Version 5.38, update May 2017; Groom *et al.*, 2016 ▸) for the substructure furan-2-carbaldehyde oxime gave 20 hits, while for substructure formaldehyde *O*-benzoyloxime there were 24 hits. The O—N distances vary from *ca* 1.38 to 1.45 Å, while the N=C distances vary from *ca* 1.25 to 1.32 Å. In the title compound, these distances are N1—O2 = 1.444 (1) Å and N1=C5 is 1.270 (2) Å, within the limits observed. In the majority of the formaldehyde *O*-benzoyloxime structures, the dihedral angle between the plane of the –COO group and the benzene ring is <10 °. In the title compound, this dihedral angle is 2.79 (16)°.

## Synthesis and crystallization   

**Synthesis of 2-furan­aldoxime:** A mixture of 5.0 g of furfuraldehyde (without further purification), 1.5 equiv. of NH_2_OH·HCl and 1 mmol of pyridine was stirred for 3 h at rt until the NH_2_OH·HCl was completely solubilized. The reaction mixture was then quenched in water and the furan­aldoxime precipitated out. This solid was filtered and recrystallized from diethyl ether to give colourless needle-like crystals (yield 4.268 g, 74%; m.p. 349–351 K). FT–IR spectrum showed two peaks at 3166 and 1634 cm^−1^. Elemental analysis: analysis calculated for C_5_H_5_NO_2_ (111.10 g mol^−1^): C, 54.05; H, 4.54; N, 12.61; O, 28.80%. Found: C, 53.13; H, 4.45; N, 12.99; O, 29.43%. ^1^H NMR (DMSO-*d*_6_): δ (ppm): 6.64 (*dd*, *J* = 3.42Hz, 0.49 Hz, 1H), 7.20 (*d*, *J* = 3.42Hz, 1H), 7.52 (*s*, 1H), 7.76 (*s*, 1H), 11.80 (*s*, 1H). ^13^C NMR (DMSO-*d*_6_): δ (ppm) = 145.85, 143.80, 135.92, 116.89, 112.67.

**Preparation of the** ***O*** **-benzoyl ester of furan­aldoxime:** Benzoyl chloride (5.01 mmol) was added dropwise under stirring to 4.55 mmol of furan­aldoxime. Since the reaction was vigorous and exothermic the mixture was placed in an ice bath for 30 min. The reaction mixture was then quenched in ice–water, and then extracted with EtOAc. The organic layer was separated and washed with 1*M* NaOH solution to remove the benzoic acid and HCl that had formed as by products. The EtOAc layer was passed through anhydrous Na_2_SO_4_ and dried *in vacuo* to give the title compound as a light-brown solid (0.9806 g). Recrystallization of the title compound from ethanol–EtOAc gave colourless needle-like crystals (yield 50%, m.p. 410–412 K). Elemental analysis: analysis calculated for C_12_H_9_NO_3_ (215.20 g mol^−1^): C, 66.97; H, 4.22; N, 6.51; O, 22.30%. Found: C, 67.00; H, 4.19; N, 6.40; O, 22.41%. ^1^H NMR (DMSO-*d*_6_): δ (ppm): 6.74–6.75 (*dd*, *J* = 3.67Hz,1.96Hz, 1H), 7.18 (*d*, *J* = 3.42Hz, 1H), 7.60 (*t*, *J* = 8.04 Hz, 2H), 7.73 (*t*, *J* = 7.58Hz, 1H), 8.01 (*s*, 1H), 8.07 (*dd*, *J* = 8.56 Hz,1.22 Hz 2H), 8.82 (*s*, 1H). ^13^C NMR (DMSO-*d*_6_): δ ppm: 163.55, 148.05, 147.65, 145.28, 134.35, 129.77, 129.48, 128.52, 119.14, 113.07.

## Refinement   

Crystal data, data collection and structure refinement details are summarized in Table 2 ▸. The H atoms were located from difference-Fourier maps and freely refined.

## Supplementary Material

Crystal structure: contains datablock(s) global, I. DOI: 10.1107/S2056989017011562/gw2156sup1.cif

Structure factors: contains datablock(s) I. DOI: 10.1107/S2056989017011562/gw2156Isup2.hkl

Supporting information file. DOI: 10.1107/S2056989017011562/gw2156Isup3.cml

CCDC reference: 1549733

Additional supporting information:  crystallographic information; 3D view; checkCIF report

## Figures and Tables

**Table 1:** Hydrogen-bond geometry (Å, °)

*D*—H⋯*A*	*D*—H	H⋯*A*	*D*⋯*A*	*D*—H⋯*A*
C8—H8⋯O2	0.93 (2)	2.384 (13)	2.724 (2)	102 (1)
C5—H5⋯O3^i^	0.97 (2)	2.312 (16)	3.159 (2)	145 (1)

Symmetry code: (i) 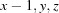.

**Table 2:** Experimental details

Crystal data	
Chemical formula	C_12_H_9_NO_3_
*M*_r_	215.2
Crystal system, space group	Monoclinic, *P*2_1_/*c*
Temperature (K)	293
*a*, *b*, *c* (Å)	6.3414 (3), 9.1268 (5), 18.1423 (9)
β (°)	95.634 (2)
*V* (Å^3^)	1044.94 (9)
*Z*	4
Radiation type	Mo *K*α
μ (mm^−1^)	0.1
Crystal size (mm)	0.19 × 0.06 × 0.04

Data collection	
Diffractometer	D8 venture
Absorption correction	Multi-scan (*SADABS*; Bruker, 2015 ▸)
*T*_min_, *T*_max_	0.87, 0.89
No. of measured, independent and observed [*I* > 3σ(*I*)] reflections	19019, 2480, 1245
*R*_int_	0.061
(sin θ/λ)_max_ (Å^−1^)	0.658

Refinement	
*R*[*F* > 3σ(*F*)], *wR*(*F*), *S*	0.037, 0.101, 1.05
No. of reflections	2480
No. of parameters	182
H-atom treatment	All H-atom parameters refined
Δρ_max_, Δρ_min_ (e Å^−3^)	0.24, −0.19

Computer programs: *APEX3* and *SAINT* (Bruker, 2015 ▸), *SIR2002* (Burla *et al.* 2003 ▸), *JANA2006* (Petricek *et al.*, 2014 ▸), *DIAMOND* (Brandenburg & Berndt, 1999 ▸) and *Mercury* (Macrae *et al.*, 2008 ▸).

## supplementary crystallographic information

### Crystal data

**Table:**

C_12_H_9_NO_3_	*F*(000) = 448
*M**_r_* = 215.2	*D*_x_ = 1.368 Mg m^−^^3^
Monoclinic, *P*2_1_/*c*	Mo *K*α radiation, λ = 0.71069 Å
Hall symbol: -P 2ybc	Cell parameters from 19019 reflections
*a* = 6.3414 (3) Å	θ = 2.3–27.9°
*b* = 9.1268 (5) Å	µ = 0.1 mm^−^^1^
*c* = 18.1423 (9) Å	*T* = 293 K
β = 95.634 (2)°	Needle, colourless
*V* = 1044.94 (9) Å^3^	0.19 × 0.06 × 0.04 mm
*Z* = 4	

### Data collection

**Table:**

D8 venture diffractometer	1245 reflections with *I* > 3σ(*I*)
Radiation source: X-ray tube	*R*_int_ = 0.061
ω and π scans	θ_max_ = 27.9°, θ_min_ = 2.3°
Absorption correction: multi-scan (SADABS; Bruker, 2015)	*h* = −7→8
*T*_min_ = 0.87, *T*_max_ = 0.89	*k* = −12→12
19019 measured reflections	*l* = −23→23
2480 independent reflections	

### Refinement

**Table:**

Refinement on *F*^2^	All H-atom parameters refined
*R*[*F* > 3σ(*F*)] = 0.037	Weighting scheme based on measured s.u.'s *w* = 1/(σ^2^(*I*) + 0.001936*I*^2^)
*wR*(*F*) = 0.101	(Δ/σ)_max_ = 0.002
*S* = 1.05	Δρ_max_ = 0.24 e Å^−^^3^
2480 reflections	Δρ_min_ = −0.19 e Å^−^^3^
182 parameters	Extinction correction: B–C type 1 Gaussian isotropic (Becker & Coppens, 1974)
0 restraints	Extinction coefficient: 8600 (1100)
0 constraints	

### Fractional atomic coordinates and isotropic or equivalent isotropic displacement parameters (Å^2^)

**Table:**

	*x*	*y*	*z*	*U*_iso_*/*U*_eq_	
O1	0.30081 (17)	0.35070 (10)	0.20660 (6)	0.0583 (4)	
O2	0.40886 (16)	0.65844 (10)	0.04205 (5)	0.0509 (4)	
O3	0.76174 (18)	0.66965 (13)	0.06946 (6)	0.0708 (5)	
N1	0.4062 (2)	0.55510 (12)	0.10223 (6)	0.0514 (5)	
C1	0.1492 (3)	0.42685 (14)	0.16340 (8)	0.0476 (5)	
C2	−0.0453 (3)	0.39050 (16)	0.18114 (10)	0.0599 (7)	
H2	−0.177 (3)	0.4294 (17)	0.1589 (9)	0.071 (5)*	
C3	−0.0152 (4)	0.28632 (18)	0.23844 (10)	0.0695 (8)	
H3	−0.120 (3)	0.2372 (18)	0.2617 (9)	0.076 (5)*	
C4	0.1916 (4)	0.26537 (18)	0.25172 (10)	0.0682 (8)	
H4	0.279 (3)	0.2027 (17)	0.2837 (9)	0.074 (5)*	
C5	0.2128 (3)	0.52772 (15)	0.10928 (8)	0.0468 (5)	
H5	0.099 (3)	0.5716 (15)	0.0768 (8)	0.060 (4)*	
C6	0.6056 (2)	0.70645 (15)	0.03182 (8)	0.0448 (5)	
C7	0.6002 (2)	0.81086 (13)	−0.03072 (7)	0.0402 (5)	
C8	0.4156 (3)	0.84781 (16)	−0.07375 (8)	0.0505 (6)	
H8	0.290 (2)	0.8040 (15)	−0.0630 (7)	0.058 (4)*	
C9	0.4214 (3)	0.94724 (17)	−0.13070 (10)	0.0600 (7)	
H9	0.295 (3)	0.9700 (17)	−0.1605 (9)	0.073 (5)*	
C10	0.6096 (3)	1.01023 (18)	−0.14491 (9)	0.0596 (7)	
H10	0.617 (3)	1.0760 (16)	−0.1852 (9)	0.069 (5)*	
C11	0.7926 (3)	0.97388 (18)	−0.10261 (9)	0.0619 (7)	
H11	0.922 (3)	1.0175 (17)	−0.1118 (9)	0.072 (5)*	
C12	0.7884 (3)	0.87486 (17)	−0.04569 (9)	0.0521 (6)	
H12	0.917 (3)	0.8530 (15)	−0.0176 (9)	0.067 (5)*	

### Atomic displacement parameters (Å^2^)

**Table:**

	*U*^11^	*U*^22^	*U*^33^	*U*^12^	*U*^13^	*U*^23^
O1	0.0656 (9)	0.0566 (6)	0.0511 (6)	−0.0020 (5)	−0.0023 (5)	0.0048 (5)
O2	0.0418 (7)	0.0557 (6)	0.0551 (6)	0.0011 (5)	0.0037 (5)	0.0127 (5)
O3	0.0408 (8)	0.0946 (8)	0.0751 (8)	0.0062 (6)	−0.0033 (6)	0.0227 (6)
N1	0.0521 (10)	0.0499 (7)	0.0518 (8)	0.0018 (6)	0.0026 (6)	0.0090 (6)
C1	0.0539 (11)	0.0431 (7)	0.0458 (9)	0.0023 (7)	0.0056 (7)	−0.0043 (6)
C2	0.0589 (13)	0.0498 (9)	0.0740 (11)	0.0015 (8)	0.0217 (10)	0.0002 (8)
C3	0.0903 (18)	0.0522 (9)	0.0718 (12)	−0.0055 (10)	0.0368 (12)	−0.0006 (9)
C4	0.1016 (19)	0.0539 (10)	0.0491 (11)	−0.0050 (11)	0.0081 (10)	0.0044 (8)
C5	0.0443 (11)	0.0464 (8)	0.0498 (9)	0.0024 (7)	0.0052 (7)	−0.0011 (7)
C6	0.0350 (10)	0.0488 (7)	0.0506 (9)	0.0034 (6)	0.0043 (7)	−0.0060 (7)
C7	0.0347 (9)	0.0416 (7)	0.0447 (8)	0.0012 (6)	0.0061 (6)	−0.0067 (6)
C8	0.0385 (11)	0.0556 (8)	0.0577 (10)	−0.0016 (7)	0.0067 (8)	0.0046 (8)
C9	0.0482 (13)	0.0682 (10)	0.0627 (11)	0.0040 (8)	0.0019 (9)	0.0134 (8)
C10	0.0586 (13)	0.0624 (10)	0.0592 (11)	−0.0018 (9)	0.0134 (9)	0.0103 (8)
C11	0.0516 (13)	0.0694 (10)	0.0668 (11)	−0.0138 (9)	0.0171 (9)	0.0000 (9)
C12	0.0373 (11)	0.0643 (9)	0.0544 (10)	−0.0023 (8)	0.0032 (8)	−0.0035 (8)

### Geometric parameters (Å, º)

**Table:**

O1—C1	1.3688 (17)	C5—H5	0.970 (15)
O1—C4	1.367 (2)	C6—C7	1.4795 (19)
O2—N1	1.4441 (14)	C7—C8	1.383 (2)
O2—C6	1.3523 (19)	C7—C12	1.379 (2)
O3—C6	1.1945 (18)	C8—H8	0.927 (16)
N1—C5	1.270 (2)	C8—C9	1.378 (2)
C1—C2	1.347 (3)	C9—H9	0.944 (17)
C1—C5	1.433 (2)	C9—C10	1.372 (3)
C2—H2	0.957 (17)	C10—H10	0.950 (16)
C2—C3	1.408 (2)	C10—C11	1.368 (2)
C3—H3	0.936 (18)	C11—H11	0.943 (17)
C3—C4	1.324 (4)	C11—C12	1.374 (2)
C4—H4	0.952 (16)	C12—H12	0.941 (16)
			
C1—O1—C4	105.29 (14)	O3—C6—C7	125.11 (14)
N1—O2—C6	113.27 (10)	C6—C7—C8	122.99 (13)
O2—N1—C5	106.73 (11)	C6—C7—C12	117.92 (13)
O1—C1—C2	110.27 (13)	C8—C7—C12	119.09 (13)
O1—C1—C5	119.30 (14)	C7—C8—H8	118.1 (8)
C2—C1—C5	130.42 (15)	C7—C8—C9	120.00 (15)
C1—C2—H2	126.0 (10)	H8—C8—C9	121.9 (9)
C1—C2—C3	106.37 (18)	C8—C9—H9	119.4 (10)
H2—C2—C3	127.6 (10)	C8—C9—C10	120.31 (16)
C2—C3—H3	127.2 (10)	H9—C9—C10	120.2 (10)
C2—C3—C4	107.0 (2)	C9—C10—H10	121.1 (10)
H3—C3—C4	125.7 (10)	C9—C10—C11	119.92 (16)
O1—C4—C3	111.09 (16)	H10—C10—C11	118.9 (10)
O1—C4—H4	114.1 (11)	C10—C11—H11	120.3 (9)
C3—C4—H4	134.8 (11)	C10—C11—C12	120.21 (17)
N1—C5—C1	122.35 (14)	H11—C11—C12	119.5 (10)
N1—C5—H5	121.6 (9)	C7—C12—C11	120.48 (15)
C1—C5—H5	116.0 (9)	C7—C12—H12	121.7 (10)
O2—C6—O3	123.68 (13)	C11—C12—H12	117.8 (10)
O2—C6—C7	111.21 (12)		

### Hydrogen-bond geometry (Å, º)

**Table:**

*D*—H···*A*	*D*—H	H···*A*	*D*···*A*	*D*—H···*A*
C8—H8···O2	0.93 (2)	2.384 (13)	2.724 (2)	102 (1)
C5—H5···O3^i^	0.97 (2)	2.312 (16)	3.159 (2)	145 (1)

Symmetry code: (i) *x*−1, *y*, *z*.

## References

[bb1] Ahluwalia, V., Kumar, J., Rana, V. S., Singh, R., Sati, O. P., Walia, S. & Garg, N. (2017). *Toxicol. Environ. Chem.* **99**, 1–9.

[bb2] Altinbas, O., Dondas, H. A., Arslan, H., Kulcu, N. & Killner, C. (2004). *Z. Kristallogr. New Cryst. Struct.* **219**, 379.

[bb3] Brandenburg, K. & Berndt, M. (1999). *DIAMOND.* Crystal Impact GbR, Bonn, Germany.

[bb4] Bruker (2015). *APEX3*, *SAINT* and *SADABS*. Bruker AXS Inc., Madison, Wisconsin, USA.

[bb5] Burla, M. C., Camalli, M., Carrozzini, B., Cascarano, G. L., Giacovazzo, C., Polidori, G. & Spagna, R. (2003). *J. Appl. Cryst.* **36**, 1103.

[bb6] Groom, C. R., Bruno, I. J., Lightfoot, M. P. & Ward, S. C. (2016). *Acta Cryst.* B**72**, 171–179. 10.1107/S2052520616003954 PMC4822653 27048719

[bb7] Harini, S. T., Kumar, H. V., Rangaswamy, J. & Naik, N. (2012). *Bioorg. Med. Chem. Lett.* **22**, 7588–7592. 10.1016/j.bmcl.2012.10.019 23116886

[bb8] Jeong, T. S., Lee, W. S., Jeong, H. J., Park, Y. D., Han, J. M., Kim, H. C., Moon, O. S. & Won, Y. S. (2013). Google patent.

[bb9] Jeong, H. J., Park, Y.-D., Park, H.-Y., Jeong, I. Y., Jeong, T.-S. & Lee, W. S. (2006). *Bioorg. Med. Chem. Lett.* **16**, 5576–5579. 10.1016/j.bmcl.2006.08.031 16919943

[bb10] Liu, X. H., Zhi, L. P., Song, B. A. & Xu, H. L. (2008). *Chem. Res. Chin. Univ.* **24**, 454–458.

[bb11] Macrae, C. F., Bruno, I. J., Chisholm, J. A., Edgington, P. R., McCabe, P., Pidcock, E., Rodriguez-Monge, L., Taylor, R., van de Streek, J. & Wood, P. A. (2008). *J. Appl. Cryst.* **41**, 466–470.

[bb12] Petricek, V., Dusek, M. & Palatinus, L. (2014). *Z. Kristallogr.* **229**, 345–352.

[bb13] Wetherington, J. B. & Moncrief, J. W. (1973). *Acta Cryst.* B**29**, 1520–1525.

